# The Benefits of COVID-19 Vaccination for Pregnant Patients Hospitalized with Respiratory Symptoms: A Retrospective Cohort Study in South Brazil

**DOI:** 10.3390/vaccines12121445

**Published:** 2024-12-22

**Authors:** Christopher J. Hernandez, Kavya G. Sundar, Fernando Echegaray, Mary Catherine Cambou, Lanbo Z. Yang, Eddy R. Segura, Marineide Gonçalves de Melo, Breno Riegel Santos, Ivana Rosângela dos Santos Varella, Karin Nielsen-Saines

**Affiliations:** 1 UCLA David Geffen School of Medicine, Los Angeles, CA 90095, USA; fechegaray@mednet.ucla.edu; 2 Cooper Medical School of Rowan University, Camden, NJ 08103, USA; kavyagsundar@gmail.com; 3 Division of Infectious Diseases, Department of Medicine, UCLA David Geffen School of Medicine, Los Angeles, CA 90095, USA; mcambou@mednet.ucla.edu; 4 Department of Obstetrics and Gynecology, Tulane University School of Medicine, New Orleans, LA 70112, USA; lanbo_yang@brown.edu; 5 Facultad de Ciencias de la Salud, Universidad Científica del Sur, Panamericana Sur Km 19, Villa, Lima 15067, Peru; eddysegura@gmail.com; 6 Hospital Nossa Senhora da Conceição, Tubarão 88701-160, Brazil; rmarineide@ghc.com.br (M.G.d.M.); breno@ghc.com.br (B.R.S.); vivana@ghc.com.br (I.R.d.S.V.); 7 Division of Pediatric Infectious Diseases, UCLA David Geffen School of Medicine, Los Angeles, CA 90095, USA; knielsen@mednet.ucla.edu

**Keywords:** SARS-CoV-2, COVID-19, vaccination, pregnancy outcomes

## Abstract

Objectives: SARS-CoV-2 infection is a known risk factor for adverse health outcomes in pregnancy, affecting both maternal and neonatal health. Mounting evidence suggests that even a single dose of an approved COVID-19 vaccine protects against severe SARS-CoV-2 infection and is safe for both pregnant persons and neonates. Southern Brazil was heavily affected by the COVID-19 pandemic, and the protective effects of the vaccine on maternal and neonatal health are not well described. This study aims to examine the protective effects of maternal COVID-19 vaccination on both maternal and neonatal outcomes following SARS-CoV-2 infection during pregnancy. Methods: This is a retrospective cohort study that leveraged medical data from a tertiary center in South Brazil to compare maternal and infant outcomes between hospitalized pregnant persons with and without SARS-CoV-2 infection between 1 March 2020, and 1 March 2023. Results: In total, 524 patients were enrolled, including 275 pregnant patients with confirmed SARS-CoV-2 infection and 249 without infection. SARS-CoV-2 infection was associated with maternal ventilator support (adjusted Risk Ratio [aRR] = 1.48, 95% Confidence Interval [95% CI]: 1.08–2.03), while receipt of at least one dose of COVID-19 vaccine was associated with protection against maternal sepsis (aRR = 0.14, 95% CI: 0.03–0.56), intensive care unit (ICU) admission (aRR = 0.27, 95% CI: 0.10–0.68), need for ventilator support (aRR = 0.60, 95% CI: 0.43–0.84), infant admission to the neonatal intensive care unit (NICU) (aRR = 0.62, 95% CI: 0.47–0.82), and neonatal respiratory distress (aRR = 0.60, 95% CI: 0.43–0.83). Conclusions: These findings further underscore the importance of maternal vaccination against COVID-19 during pregnancy. Even one dose of vaccine was protective against a variety of maternal and neonatal outcomes. Prenatal care should encourage COVID-19 vaccination in pregnancy.

## 1. Introduction

Brazil holds one of the highest total mortalities due to COVID-19, with geographically distinct patterns of incidence, morbidity, and mortality rates [1]. The state of Rio Grande do Sul, for example, reached an incidence rate of 4472 per 100,000 people, an incidence higher than the national average of 3129 per 100,000 people. However, the mortality rate of the state was lower, standing at 81 per 100,000 compared to the national average of 84 per 100,000 [1]. Despite this, Rio Grande do Sul and the state capital of Porto Alegre had the highest total mortality among the southern states of Brazil [2]. Brazil also documented an exceedingly high mortality figure among pregnant and post-partum individuals. Reflecting this, the maternal mortality rate increased among all states in Brazil by the end of 2021, with the south holding a lower maternal mortality rate compared to other regions in Brazil [3]. As of 2022, Rio Grande do Sul has the 6th highest human development index across the 26 states of Brazil, which may have translated into more prompt access to higher quality healthcare among pregnant people with COVID-19 [4]. This underscores the importance of describing the regional patterns of COVID-19 incidence, vaccine uptake, and potential associated socio-demographics.

Pregnancy is a well-documented risk factor for severe and critical COVID-19 [5]. Compared with non-pregnant patients, higher rates of ICU admission and requirements for invasive ventilation have been observed among pregnant women who are hospitalized with SARS-CoV-2 infection [6]. Normal physiological changes during pregnancy, including changes to the immune system, increased cardiovascular requirements, and decreased lung capacity potentially contribute to the increased susceptibility to severe disease, evidenced by a greater risk for adverse outcomes during the later stages of pregnancy, when the most profound changes take place [7]. Further, COVID-19 has been associated with greater rates of pregnancy complications. Compared to pregnant women with asymptomatic or mild SARS-CoV-2 infection, women with severe COVID-19 have been shown to have increased rates of preterm delivery [8,9]. In addition, a study of 200 pregnant women with SARS-CoV-2 infection reported that a large number of neonates born to mothers with moderate to severe COVID-19 were admitted to the neonatal intensive care unit (NICU) [10].

COVID-19 vaccination has been shown to be a safe and effective way to prevent severe COVID-19 in pregnancy, although uptake among pregnant persons in Brazil remains subpar due to fear surrounding both maternal and neonatal side effects. A study in Rio de Janeiro, Brazil examining 17,513 live births between 15 May 2021 and 23 October 2021 demonstrated that maternal COVID-19 vaccination was safe, as it was not associated with preterm birth, small for gestational age events, low birth weight, low Apgar scores, or death [11]. In terms of efficacy, a prospective cohort study conducted in Rio de Janeiro found that mothers who were vaccinated with at least one dose were significantly less likely to have severe morbidity and mortality associated with SARS-CoV-2 infection [12]. A systematic review of 30 studies involving 862,272 pregnant patients (308,428 vaccinated and 553,844 unvaccinated) found that COVID-19 vaccination during pregnancy reduced COVID-19 disease severity and was not associated with any adverse maternal and neonatal outcomes [13]. As such, the current guidelines set out by the Brazilian Obstetrics Society and the American College of Obstetrics and Gynecologists recommend that all pregnant patients complete the vaccination series at any point during pregnancy [14,15].

The objective of the present study was twofold: (1) to analyze the associations of adverse maternal and neonatal outcomes in pregnant patients hospitalized with respiratory symptoms with and without SARS-CoV-2 infection in a cohort of pregnant patients in south Brazil, and (2) to estimate the uptake and protective effects of COVID-19 vaccination among this cohort. A significant novelty of this study is the control population, which includes pregnant people with respiratory symptoms that could be explained by viruses other than SARS-CoV-2, which may also be associated with adverse maternal outcomes.

## 2. Methods

### 2.1. Study Site

This retrospective cohort study spanning 1 March 2020 to 1 March 2023 was performed at a tertiary healthcare center in South Brazil that provides specialized care for medium- and high-risk pregnancies in a low-income region of the city of Porto Alegre, in the state of Rio Grande do Sul. Approximately 3500 deliveries occur at this institution per year.

### 2.2. Patient Selection and Data Sources

This is a retrospective cohort study of pregnant patients at a reference institution who were tested for SARS-CoV-2 infection. Patients had to be hospitalized at this institution at any point during their pregnancy and had to be reported to one of the two notification systems during their hospital stay (either the SIVEP-Gripe or E-SUS Notifica system) [16,17]. There were two possible timepoints at which patient medical records and clinical information were accessed. The first timepoint, and not mandatory for inclusion, was the date of hospitalization for any obstetric or clinical reason prior to delivery that was reported to the SIVEP-Gripe or E-SUS Notifica systems. The second timepoint, and mandatory for inclusion in this study, was at the time of delivery. The hospitalization for delivery had to be reported via SIVEP-Gripe or E-SUS Notifica if the patients did not have a previously notified hospitalization during that pregnancy. That is, patients had to be reported to SIVEP-Gripe or E-SUS Notifica at least once during pregnancy: (1) during a hospitalization prior to delivery or (2) at the time of delivery. If a patient had more than one hospitalization during pregnancy recorded in the notification systems, we chose data from the hospitalization associated with a laboratory-confirmed SARS-CoV-2 infection. If all SARS-CoV-2 tests were negative among patients with respiratory symptoms, we chose the hospitalization closest to delivery. Nasopharyngeal specimen testing for SARS-CoV-2 real-time reverse transcription polymerase chain reaction (RT-PCR) or a rapid antigen detection test were used to confirm infection. Patients who met these criteria but did not deliver at the study site were excluded because we did not have access to the outcome information regarding their delivery and post-partum period.

To calculate the sample sizes needed for this study, we assumed a two-sided significance level of 0.05, a power (1-beta) level of 0.8, an approximate ratio of unexposed to exposed of 1:1, a baseline probability of adverse events in the unexposed group of 0.01, and a relative risk of adverse outcomes for those exposed to SARS-CoV-2 infection of approximately 5 [8,9]. Based on this, we calculated an approximate requirement of 285 patients per group.

The SIVEP-Gripe and E-SUS Notifica systems are public surveillance databases for respiratory infections and notifiable diseases from the Brazilian Unified Single Health System (SUS) to which medical institutions of the Single Unified Health System report to, as in the case of our institution. The two notification systems were used prior to March 1st, 2021: only pregnant patients with symptoms of respiratory infection were routinely tested for COVID-19, potentially leaving out asymptomatic SARS-CoV-2 cases from the database. After the emergence of the gamma variant in Brazil in January 2021, guidelines to test all pregnant patients for SARS-CoV-2 infection were put in place. The SIVEP-Gripe holds demographic and clinical history for all patients hospitalized with respiratory symptoms or acute respiratory distress syndrome (ARDS) regardless of SARS-CoV-2 infection [16]. The E-SUS Notifica system includes demographic and clinical history for patients with any respiratory symptoms during hospitalization, whether they were SARS-CoV-2 positive or negative, or if they had asymptomatic SARS-CoV-2 infection [17].

### 2.3. COVID-19 Vaccination

In March 2021, the Brazilian Ministry of Health authorized SARS-CoV-2 vaccination for pregnant women with comorbidities. Initially, the CoronaVac (whole inactivated SARS-CoV-2, Sinovac Biotech/Butantan Institute, São Paulo, Brazil), the AstraZeneca adenovirus viral vector vaccine (Oxford/Biomanguinhos, Rio de Janeiro, Brazil), Janssen adenovirus viral vector vaccine (Johnson and Johnson/Janssen Pharmaceuticals, Leiden, The Netherlands) and the BNT162b2 mRNA vaccine (Pfizer-BioNTech, Kalamazoo, MI, USA) were authorized for use in Brazil. In mid-May 2021, the Ministry of Health halted administration of the AstraZeneca vaccine to pregnant women due to a cardiovascular accident event that occurred in a pregnant patient after vaccination [18]. In late June 2021, COVID-19 vaccination was made available to all pregnant women in Brazil, regardless of comorbid conditions. In September 2021, the Ministry of Health recommended that pregnant women should receive either BNT162b2 or CoronaVac.

### 2.4. Demographic and Clinical Measurements

The co-variates analyzed were age, race, geographical region of residence, trimester of pregnancy at time of hospitalization, caesarean delivery, multiparity, maternal comorbidities, and history of at least one dose of the COVID-19 vaccine. These variables were selected because they are known causes of maternal mortality in low-income countries, and because they have been investigated in previous studies of risk factors for severe morbidity and mortality among women with SARS-CoV-2 infection during pregnancy [19].

### 2.5. Clinical Outcomes

The primary outcomes related to severe maternal morbidity were defined as ICU admission or need for ventilator support. Pregnancy outcomes included hypertensive disorders of pregnancy, maternal sepsis, postpartum hemorrhage, preterm birth (defined as gestational age at time of delivery less than 37 weeks), stillbirth (>=20 weeks), or miscarriage (<20 weeks). Maternal mortality, defined as death during the hospitalization or up to 42 days postpartum, was described; however, all instances of mortality occurred among patients who were admitted to the ICU and were considered within that outcome in the analysis. Neonatal outcomes included birthweight, NICU admission, or neonatal respiratory distress.

### 2.6. Statistical Analysis

Descriptive statistics were reported for demographic and clinical data. Bivariate associations between SARS-CoV-2 infection and demographic, clinical data, and selected maternal and neonatal outcomes were assessed by conducting Chi-square tests of independence comparing categorical (e.g., SARS-CoV-2 infection distribution differences among rural vs. urban vs. greater metropolitan region) or dichotomous (e.g., SARS-CoV-2 infection distribution differences among smokers vs. non-smokers) variable exposures. Bivariate modified Poisson regressions between outcomes of interest and SARS-CoV-2 infection were conducted to assess magnitude of association [20]. A multivariable modified Poisson regression analysis was performed for each maternal or neonatal clinical outcome of interest, with the primary exposure variables being SARS-CoV-2 infection during hospitalization and receipt of at least one dose of COVID-19 vaccine, further controlling for age, parity, gestational age, and composite score of preexisting comorbidities [21]. Models were checked for model fitness and collinearity. Significant independent associations between the maternal or neonatal clinical outcome of interest and SARS-CoV-2 infection or receipt of at least one dose of COVID-19 vaccine were reported. Patients with missing data or who did not have information for a given outcome were excluded from the pertinent models. A sensitivity analysis looking at two doses of vaccination was conducted following same steps as above. Statistical analysis was performed using STATA 14.

## 3. Results

### 3.1. Socio-Demographics

**Figure 1:**
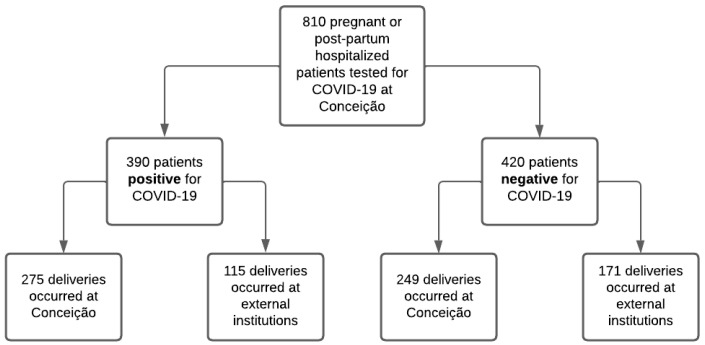
Patient selection cascade among SARS-CoV-2 positive and negative pregnant patients.

In total, 810 pregnant or immediately post-partum patients hospitalized at any time during pregnancy at the participating institution and tested for SARS-CoV-2 infection were potential study candidates. Among these, 390 participants tested positive for SARS-CoV-2 and 420 tested negative (Figure 1). In total, 524 participants were ultimately enrolled in the study because they delivered at the participating institution and had primary outcome data available. This included 275 participants with laboratory-confirmed SARS-CoV-2 infection during pregnancy and 249 who tested negative. Among participants delivering at the tertiary healthcare center, most (76%) were between 18 and 34 years of age, 20% were 35 years of age or over, and a minority (4%) were under the age of 18 (Table 1). The majority of participants (68%) identified as White, a fifth (20%) identified as Black, and 13% identified as multiracial. Over half (51%) of patients had completed less than high school, 41% completed high school, 8% had a university degree, and less than 1% did not report level of education. Most patients (72%) resided in the urban region of Porto Alegre, while 24% came from the greater metropolitan region, and 4% from a rural region. The most common preexisting comorbidities included obesity at 22%, hypertension at 19%, and present or history of smoking at 18%. Most (66%) patients were multigravida and 46% underwent a C-section during delivery.

#### 3.1.1. SARS-CoV-2 Infection

Among participants with laboratory-confirmed SARS-CoV-2 infection, 22% were asymptomatic, 51% had mild to moderate disease, and 27% had severe to critical disease (Supplementary Table S1). Fifty percent of those with mild to moderate disease compared to 77% of those with severe to critical disease had had not received a single dose of the COVID-19 vaccine (*p* < 0.001).

#### 3.1.2. COVID-19 Vaccination

Forty-eight percent of the cohort had received at least one dose of the COVID-19 vaccine at the time of hospitalization. Among those vaccinated (n = 252), the most common vaccine used was BNT162b2 mRNA vaccine (Pfizer-BioNTech, Kalamazoo, MI, USA) at 68.3%, followed by CoronaVac (whole inactivated SARS-CoV-2, Sinovac Biotech/Butantan Institute, São Paulo, Brazil), at 19.0 AstraZeneca adenovirus viral vector vaccine (Oxford/Biomanguinhos, Rio de Janeiro, Brazil), at 11.5%, and the Janssen adenovirus viral vector vaccine (Johnson and Johnson/Janssen Pharmaceuticals, Leiden, The Netherlands) at 1.2%.

### 3.2. Bivariate Associations

Socio-economic factors associated with SARS-CoV-2 infection included region of residence, with higher positivity seen in patients coming from rural regions (7% positives vs. 1%, negatives *p* = 0.001) or the greater metropolitan area (26% positives vs. 22% negatives, *p* = 0.001), while in urban areas, a higher frequency of negative patients was seen (67% positives vs. 77% negatives, *p* = 0.0001), likely reflective of hospital referral patterns. Interestingly, SARS-CoV-2 positive patients were less likely to smoke than negative patients (12% vs. 24%, *p* = 0.001; Table 1). Further, maternal ICU admission (9% vs. 4%, *p* = 0.048), ventilator support (27 vs. 18%, *p* = 0.005), and NICU admission (36% vs. 27%, *p* = 0.026) were associated with SARS-CoV-2 infection (Table 2). There were five cases of maternal mortality, and all occurred among patients who were admitted to the ICU. Four deaths occurred among those with SARS-CoV-2, and one occurred among SARS-CoV-2 negative patients.

### 3.3. Multivariable Associations

At the multivariable level (Table 3), ventilator support was found to be independently associated with SARS-CoV-2 infection (adjusted Risk Ratio [aRR] = 1.48, 95% Confidence Interval [95% CI] = 1.08–2.03). We found that receipt of at least one dose of COVID-19 vaccination was associated with lower risk of maternal sepsis (aRR = 0.14, 95% CI 0.03–0.56), lower risk of ICU admission (aRR = 0.27, 95% CI 0.10–0.68), lower risk of ventilator support (aRR = 0.60, 95% CI 0.43–0.84), lower risk of NICU admission (aRR = 0.62, 95% CI 0.47–0.82), and lower risk of neonatal respiratory distress (aRR = 0.60, 95% CI 0.43–0.83). In the sensitivity analysis, receipt of at least two doses of COVID-19 vaccination was largely comparable to receipt of one vaccine dose (Supplementary Table S2).

## 4. Discussion

We found important associations between adverse maternal and neonatal outcomes and maternal SARS-CoV-2 infections during pregnancy among hospitalized pregnant persons at a large tertiary healthcare center in south Brazil. Pregnant patients who were admitted to the ICU or required ventilator support were more likely to have been diagnosed with SARS-CoV-2 infection; however, only ventilator support was independently associated with SARS-CoV-2 infection. We found that slightly less than half of patients had at least one dose of COVID-19 vaccination, with vaccination being associated with a significantly reduced risk for multiple maternal and neonatal outcomes. Most patients in this study did not have COVID-19 vaccination at the time of hospitalization, and accounted for the greatest proportion of those who presented with mild to moderate or severe to critical SARS-CoV-2 disease severity. Maternal vaccination was independently associated with a significant risk reduction for maternal sepsis, ICU admission, and need for ventilator support. Maternal receipt of at least one COVID-19 vaccine dose protected against NICU admission and neonatal respiratory distress. C-sections in this study were high, consistent with studies that suggest Brazil is one of the countries with the highest rate of C-sections in the world [22].

Our findings are broadly consistent with multiple studies that have noted increased rates of adverse maternal outcomes among hospitalized pregnant women with SARS-CoV-2 infection [23,24]. SARS-CoV-2 infection in pregnancy can lead to worsened outcomes, as the natural physiological, endocrinological, and immunological changes associated with pregnancy may predispose patients to respiratory failure in the setting of certain viral infections, including influenza [25,26]. In fact, a metanalysis study found that compared to women who were not pregnant, pregnant women with SARS-CoV-2 infection were found to have increased rates of ICU admission and requirements for invasive mechanical intervention, further highlighting the predisposition to acute adverse outcomes in pregnancy [6].

We found that maternal infection was associated with NICU admission, even though vertical transmission rates of SARS-CoV-2 are generally very low, estimated to be less than 2% [27]. Changes to the maternal inflammatory profile seen in SARS-CoV-2 infection during pregnancy may explain this association. A recent study found that neonates born to mothers with SARS-CoV-2 infection in pregnancy had a similar immune profile to adults living with COPD in the absence of vertical transmission of SARS-CoV-2 to the neonate [10]. An earlier study from our group identified significant inflammatory pathways in pregnant mothers and their newborn infants, despite none of the latter being infected with SARS-CoV-2 and inflammatory cytokines and chemokines not crossing the placenta and being unique in exposed infants [28]. Further, a study examining pathological changes to the placenta of mothers infected with SARS-CoV-2 during the third trimester found increased features of abnormal maternal vascular perfusion, suggesting a separate mechanism by which neonatal outcomes could be altered, although in our study, trimester of infection was not found to have a significant association with infant respiratory distress [29]. Regardless, these data add to a body of growing literature that implicates maternal SARS-CoV-2 infection in the development of adverse neonatal outcomes and highlights the need to increase prevention and early management of SARS-CoV-2 infection during pregnancy to optimize outcomes.

Interestingly, although maternal SARS-CoV-2 infection rates did not differ based on maternal vaccination status, our study found that vaccination was protective against multiple adverse maternal and neonatal outcomes. Consistent with the US Centers for Disease Control, we found that vaccination does not necessarily prevent infection, but does protect against severe disease [30]. Studies have found that waning humoral immunity and viral evolution may allow for viral escape, but anamnestic T cell responses to conserved viral epitopes prevent severe infection [31]. As such, our data are consistent with a longitudinal cohort study in Rio de Janeiro which found that the risk for severe maternal morbidity and mortality was significantly reduced when patients were vaccinated [12]. Similarly, maternal sepsis, which arises when an immune response towards an infection or toxin is dysregulated [32], was less likely to occur among pregnant patients who were vaccinated. Vaccination may have provided a more measured immune response to SARS-CoV-2 infection, which reduced the inflammatory burden and lowered the risk for meeting criteria for sepsis. Consistent with the study as well, we found that maternal vaccination rates were suboptimal, which was due to the lack of availability of vaccines and lack of early guidelines recommending immunization of pregnant women. Vaccines only became available to most pregnant patients in April of 2021, with more prominent immunization of pregnant women occurring in the second half of 2021. We found, however, that maternal vaccination not only confers protection to the mother, but also to the neonate, and was not related to any adverse maternal or neonatal outcomes in our study. Vaccination may confer protection to the neonate by preventing aberrant changes to the cytokine profile as discussed above, leading to reduced rates of respiratory complications. Transplacental transfer of maternal anti-SARS-CoV-2 IgG antibodies is significantly enhanced after vaccination (hybrid immunity), which could also play a protective role for the neonate, conferring protection until the infant is at least 6 months of age and then able to be immunized [33,34]. Further, a study found that maternal vaccination with two doses of mRNA vaccine was associated with a reduced risk of hospitalization for COVID-19, including for critical illness, among infants younger than 6 months of age [35]. These results indicate that, in this population, vaccination against COVID-19 is a safe and effective strategy to reduce maternal and neonatal morbidity.

## 5. Limitations

There are important limitations to consider when examining these data. The first limitation is the retrospective nature of the study, with data derived from patients who were hospitalized for any reason at some point during pregnancy. The guidelines set by the Brazilian Ministry of Health evolved throughout the pandemic as new understandings emerged, changing the case definitions of symptomatic disease. Due to temporal changes in SARS-CoV-2 screening, there were likely eligible patients who were not reported to the SIVEP-Gripe notification system. This is particularly true from the start of the pandemic to March 2021, when only pregnant women with respiratory symptoms were tested for SARS-CoV-2 infection, likely leading to missed asymptomatic diagnoses. Similarly, we were limited to SARS-CoV-2 screening tests provided when women were hospitalized, often at the time of delivery. We could not determine if there were SARS-CoV-2 screens or if there was COVID-19 disease at home throughout their pregnancy. These two limitations may affect the internal validity of this study, as pregnant woman who did have SARS-CoV-2 infection were not categorized as exposed. We were also unable to control for a previous SARS-CoV-2 infection, which may have conferred a degree of protection against subsequent disease severity. We were unable to conduct a more granular analysis comparing the various types of vaccinations that were provided because of the small sample size, and had to collapse vaccination categories into vaccinated vs. unvaccinated. The temporal spread of SARS-CoV-2 and COVID-19 vaccination in this study presents further limitations. The earlier period had a largely naïve population that was not exposed to SARS-CoV-2 through infection or vaccine, which could have disproportionately affected health outcomes. Survivor bias in the later years, such as late 2022 or early 2023, could mean that adverse outcomes in this time period were less frequent as more and more people gained immunity through infection or vaccine. Finally, the nature of our population, pregnant women being served at a public healthcare institution in south Brazil, may overrepresent women of lower socio-economic status facing unique structural, economic, social, and cultural contexts that limit the generalizability of this study to other populations.

## 6. Conclusions

This study among hospitalized pregnant patients with respiratory symptoms further characterizes the impact of SARS-CoV-2 infection in pregnancy compared to other respiratory infections and highlights that maternal receipt of at least one dose of a COVID-19 vaccine was protective against multiple adverse maternal and neonatal outcomes. Maternal vaccination against SARS-CoV-2 was again proven to be very safe and extremely effective in the reduction of maternal and neonatal morbidity and mortality risk, highlighting the need for this practice to be highly encouraged in pregnant patients.

## Figures and Tables

**Table 1:** Maternal and infant demographics and clinical history for pregnancies evaluated for SARS-CoV-2 during hospitalization (during pregnancy or in the post-partum period) between March 2020 and March 2023.

	All Pregnancies *n* = 524 (%)	SARS-CoV-2 Positive *n* = 275 (%)	SARS-CoV-2 Negative *n* = 249 (%)	*p*-Value ^a^
Age at time of hospitalization				
<18	19 (3.6)	7 (2.6)	12 (4.8)	0.110 ^b^
18–34	398 (76.0)	204 (74.2)	194 (77.9)	
≥35	107 (20.4)	64 (23.3)	43 (17.3)	
Region				
Urban	375 (71.6)	184 (66.9)	191 (76.7)	0.001 ^b^
Greater metropolitan region	126 (24.4)	71 (25.8)	55 (22.1)	
Rural region/outskirts	23 (4.4)	20 (7.3)	3 (1.2)	
Race				
Black	103 (19.7)	61 (22.2)	42 (16.9)	0.267 ^b^
Multiracial	67 (12.8)	32 (11.6)	35 (14.1)	
White	354 (67.6)	182 (66.2)	172 (69.1)	
Education level				
None or Unknown	4 (0.8)	1 (0.4)	3 (1.2)	0.085 ^b^
Less than high school	266 (50.8)	130 (47.3)	136 (54.6)	
High school completion	214 (40.8)	117 (42.6)	97 (39.0)	
University completion	40 (7.6)	27 (9.8)	13 (5.2)	
Type of delivery				
Vaginal	264 (50.4)	137 (52.5)	127 (52.5)	0.998 ^b^
C-section	239 (45.6)	124 (47.5)	115 (47.5)	
Gravida				
Primigravid	178 (34.0)	96 (34.9)	82 (32.9)	0.633 ^b^
Multigravid	346 (66.0)	179 (65.1)	167 (67.1)	
Twin gestation	13 (2.5)	7 (2.7)	6 (2.5)	0.886 ^c^
Maternal Comorbidities				
Pre-existing systems disorders	49 (19.3)	24 (8.7)	25 (10.0)	0.606 ^c^
Immunodeficiencies + HIV	21 (4.0)	12 (4.4)	9 (3.6)	0.662 ^c^
Pulmonary disease + asthma	74 (14.1)	36 (13.1)	38 (15.3)	0.476 ^c^
Obesity (pre-pregnancy BMI > 30)	114 (21.8)	59 (21.5)	55 (22.1)	0.861 ^c^
Hypertension (pre-existing)	99 (18.9)	53 (19.3)	46 (18.5)	0.816 ^c^
Diabetes (pre-existing)	15 (2.9)	10 (3.6)	5 (2.0)	0.264 ^c^
Smoking (current or Hx)	96 (18.3)	34 (12.4)	62 (24.9)	0.001 ^c^
COVID-19 vaccination doses				
No vaccination	272 (51.9)	148 (53.8)	124 (49.8)	0.358 ^c^
At least 1 vaccination	252 (48.1)	127 (46.2)	125 (50.2)	
Vaccine type (first and/or second dose) ^d^				
Pfizer	172 (68.3)	85 (67.0)	87 (69.6)	0.520 ^b^
Sinovac	48 (19.0)	22 (17.3)	26 (20.8)	
Johnson and Johnson	3 (1.2)	2 (1.6)	1 (0.8)	
Oxford-AstraZeneca Covishield	29 (11.5)	18 (14.1)	11 (8.8)	

a. Bivariate associations between socio-demographics and clinical outcomes with SARS-CoV-2 infection were assessed through Chi-square tests of independence. b. Comparisons conducted among categorical variable (i.e., belongs to one of the groups) distributions of SARS-CoV-2 exposed group versus non-exposed groups. c. Comparisons conducted between dichotomous variable (i.e., no versus yes for the characteristic) distributions of SARS-CoV-2 exposed group versus non-exposed groups. d. Among those vaccinated (n = 252).

**Table 2:** Selected maternal and infant outcomes during hospitalization (during pregnancy or in the post-partum period) between March 2020 and March 2023.

	All Pregnancies *n* = 524 (%)	SARS-CoV-2 Positive *n* = 275 (%)	SARS-CoV-2 Negative *n* = 249 (%)	*p*-Value ^a^
Hypertensive disorders in pregnancy	171 (32.6)	82 (29.8)	89 (35.7)	0.149 ^c^
Postpartum hemorrhage	73 (13.9)	36 (13.1)	37 (14.9)	0.559 ^c^
Maternal sepsis	23 (4.4)	11 (4.0)	12 (4.8)	0.648 ^c^
Gestational age at time of delivery (n = 532)				
<37 weeks	130 (24.8)	72 (27.6)	58 (24.0)	0.354 ^b^
37–41 weeks	373 (71.2)	189 (72.4)	184 (76.0)	
Birth outcomes (n = 553)				
Stillbirths’	11 (2.1)	6 (2.2)	5 (2.0)	0.407 ^b^
Miscarriages (<20 weeks)	21 (4.0)	14 (5.1)	7 (2.8)	
Live birth	492 (93.9)	255 (92.7)	237 (95.2)	
Maternal outcomes				
ICU admission (yes/no)	35 (6.7)	24 (8.7)	11 (4.4)	0.048 ^c^
Ventilator support				
No support	402 (76.7)	199 (72.4)	203 (81.5)	0.005 ^b^
Non-invasive	93 (17.7)	52 (18.9)	41 (16.5)	
Invasive	26 (5.0)	22 (8.0)	4 (1.6)	
Maternal Death	5 (1.0)	4 (1.5)	1 (0.4)	0.216 ^c^
Neonatal outcomes				
Fetal sex (n = 515)				
Male	279 (54.2)	143 (53.4)	136 (55.1)	0.698 ^b^
Female	236 (45.8)	125 (46.6)	111 (44.9)	
NICU-admission (n = 503)	157 (31.2)	93 (35.6)	64 (26.5)	0.026 ^c^
Neonatal Respiratory Distress (n = 503)	123 (25.1)	68 (26.1)	58 (23.9)	0.589 ^c^
Size for gestational age (n = 499)				
Appropriate growth	380 (76.2)	200 (77.5)	180 (74.7)	0.458 ^b^
SGA/LGA	119 (23.8)	58 (22.5)	61 (25.3)	

a. Bivariate associations between selected maternal and infant outcomes with SARS-CoV-2 infection were assessed through Chi-square tests of independence. b. Comparisons conducted among categorical variable (i.e., belongs to one of the groups) distributions of SARS-CoV-2 exposed group versus non-exposed groups. c. Comparisons conducted between dichotomous variable (i.e., no versus yes for the characteristic) distributions of SARS-CoV-2 exposed group versus non-exposed groups.

**Table 3:** Associations between maternal SARS-CoV-2 infection and at receipt of at least one maternal SARS-CoV-2 vaccination and selected outcomes. Significant adjusted RR for SARS-CoV-2 infection and SARS-CoV-2 vaccination are shown.

Outcome	Unadjusted RR (95% CI) Exposure: SARS-CoV-2 Infection	Adjusted RR (95% CI) Exposure: SARS-CoV-2 Infection	Unadjusted RR (95% CI) Exposure: At Least One COVID-19 Vaccine Dose	Adjusted RR (95% CI) Exposure: At Least One COVID-19 Vaccine Dose
Hypertensive disorders in pregnancy	0.87 (0.73–1.05)		1.19 (0.99–1.42)	
Postpartum hemorrhage	0.93 (0.73–1.19)		0.77 (0.57–1.05)	
Maternal sepsis	0.91 (0.59–1.40)		0.17 (0.46–0.66)	0.14 (0.03–0.56)
Preterm birth	1.09 (0.91–1.31)		1.31 (1.04–1.66)	
Fetal demise	1.21 (0.91–1.60)		0.77 (0.49–1.21)	
ICU admission	1.34 (1.05–1.70)		0.28 (0.12–0.64)	0.27 (0.10–0.68)
Maternal Ventilator support	1.23 (1.06–1.49)	1.48 (1.08–2.03)	0.62 (0.47–0.82)	0.60 (0.43–0.84)
Birthweight (SGA or LGA)	0.93 (0.75–1.14)		0.83 (0.65–1.06)	
NICU-admission (n = 520)	1.22 (1.03–1.45)		0.65 (0.52–0.82)	0.62 (0.47–0.82)
Neonatal Respiratory Distress (n = 520)	1.05 (0.87–1.27)		0.65 (0.51–0.85)	0.60 (0.43–0.83)

SARS-CoV-2 vaccination was defined as having at least 1 dose of vaccine administered prior to hospitalization. Each outcome of interest was analyzed in individual multivariate models, controlling for age, parity, gestational age, preexisting comorbidities, as well as the exposures of interest (i.e., SARS-CoV-2 infection and COVID-19 vaccination). Adjusted RRs for SARS-CoV-2 infection and COVID-19 vaccination are derived from each of the models.

## Data Availability

The data presented in this study are available on request from the corresponding author due to confidentiality concerns as this was a relatively small sample of PPLH in a specific region.

## Supplementary Materials

The following supporting information can be downloaded at: https://www.mdpi.com/article/10.3390/vaccines12121445/s1, Table S1: Association between COVID-19 disease severity and receipt of at least one dose of SARS-CoV-2 vaccine. Table S2: Selected outcomes and their association with maternal reciept of at least two COVID-19 vaccines at the time of hospitalization.
