# Supplementary material for: The Benefits of COVID-19 Vaccination for Pregnant Patients Hospitalized with Respiratory Symptoms: A Retrospective Cohort Study in South Brazil
Source: Vaccines (Basel). 2024 Dec 22;12(12):1445. doi: 10.3390/vaccines12121445 (PMC11680175; doi:10.3390/vaccines12121445)
Supplement: Supplementary file 1 [file vaccines-12-01445-s001.zip › vaccines-3318217-supplementary.pdf]

Supplemental Table S1. Association between COVID-19 disease severity and receipt of at least one dose of SARS-CoV-2 vaccine.

| COVID-19 disease severity | No vaccination | Vaccinated at least 1 time | Chi-square p-value |
|---|---|---|---|
| Asymptomatic (n = 60) | 21 (35.0) | 39 (65.0) | 0.001 |
| Mild/Moderate (n = 141) | 70 (49.7) | 71 (50.4) | |
| Severe/Critical (n = 74) | 57 (77.0) | 17 (23.0) | |

Supplementary Table S2. Selected outcomes and their association with maternal reciept of at least two COVID-19 vaccines at the time of hospitalization.

| Outcome | Unadjusted RR (95% CI) Exposure: at least two COVID-19 vaccine doses | Adjusted RR (95% CI) Exposure: at least two COVID-19 vaccine doses |
|---|---|---|
| Hypertensive disorders in pregnancy | 1.10 (0.86 – 1.40) | |
| Postpartum hemorrhage | 0.71 (0.47 – 1.07) | |
| Maternal sepsis | 0.12 (0.02 – 0.81) | 0.10 (0.01 – 0.69) |
| Preterm birth | 0.81 (0.60 – 1.08) | |
| Fetal demise | 0.70 (0.38 – 1.29) | |
| ICU admission/death | 0.23 (0.08 – 0.69) | 0.24 (0.07 – 0.78) |
| Maternal Ventilator support | 0.51 (0.35 – 0.74) | 0.53 (0.35 – 0.81) |
| Birthweight (SGA or LGA) | 0.92 (0.68 – 1.25) | |
| NICU-admission (n=520) | 0.68 (0.51 – 0.91) | 0.74 (0.52 – 0.96) |
| Neonatal Respiratory Distress (n=520) | 0.66 (0.47 – 0.91) | 0.37 (0.21 – 0.67) |

SARS-CoV-2 vaccination was defined as having at least 2 doses of vaccine administered prior to hospitalization. Each outcome of interest was analyzed in individual multivariate models, controlling for age, parity, gestational age, preexisting comorbidities, as well as the exposures of interest (i.e. SARS-CoV-2 infection and COVID-19 vaccination).
